# Impact of orientation specific surround modulation and tuning curve shape on population coding and tilt illusion in V1

**DOI:** 10.1186/1471-2202-14-S1-P404

**Published:** 2013-07-08

**Authors:** Sander W Keemink, Clemens Boucsein, Mark CW van Rossum

**Affiliations:** 1 School of Informatics, University of Edinburgh, Edinburgh, EH8 9AB, UK; 2 Bernstein Center Freiburg, University of Freiburg, Freiburg, 79104, Germany

## 

The tilt illusion is a well-studied visual phenomenon, whereby the perceived angle of a center stimulus is misjudged in the presence of a differently aligned surround stimulus (e.g. [1]). The dependence of V1 neuron activity on center-surround interactions has been studied extensively (e.g. [2]). These center-surround interactions can be used to explain the tilt illusion, as they result in tuning curve modulations. When population activity is decoded using these modulated tuning curves, the tilt illusion arises [2]. In this work, we examine two factors affecting the tilt illusion:

First, we examine is the effect of the tuning curve width on the tilt illusion. Tuning curves widths vary widely in vivo [3]. Although changes in tuning curve width due to center-surround interactions have been shown to potentially contribute to the tilt illusion [2,4], how the tuning curve width itself affects the illusion is less well understood. Using a firing rate model, we show here that for narrower tuning curves the tilt illusion lessens, and that it disappears almost completely for narrow, but still realistic, tuning curves.

Secondly, we consider the consequences of recent experimental findings on the tuning of surround modulation. Most models assume that V1 neurons experience most suppression when the surround stimulus is aligned with the neuron's preferred orientation. However, a recent study showed that for the majority of V1 neurons, the suppression effect depends much more on the relation between center and surround orientation, being strongest when they are co-aligned, regardless of the preferred orientation [5]. We use a firing rate model based on [5] to take these new finding into account, and show that, counter-intuitively, the tilt illusion is not impacted, once we control for changes in the tuning curve widths.
